# Supplementary material for: An explicit integration approach for predicting the microstructures of multicomponent alloys
Source: Nat Commun. 2025 Jul 15;16:6504. doi: 10.1038/s41467-025-61246-7 (PMC12264088; doi:10.1038/s41467-025-61246-7)
Supplement: Supplementary file 1 — Supplementary Information [file 41467_2025_61246_MOESM1_ESM.pdf]

Supplementary information for: An explicit integration approach for predicting the microstructures of multicomponent alloys

Supplementary Note 1: Estimated computation time for solving internal equilibrium conditions using PyCalphad

In a 12-component system, solving the internal equilibrium condition for the γ' phase in the superalloy took 0.30 s using the CALPAHD software PyCalphad. This calculation speed is sufficiently fast to generate a phase diagram, which is the main purpose of the CALPHAD method; however, it is too slow to be used for phase-field simulations, because in phase-field simulations, the internal equilibrium condition needs to be solved at every computational grid point where the sublattice phase exists and at every timestep. For example, when performing the calculation for Fig. 1 d, the internal equilibrium condition must be solved 2.5×10^8 times (128 grids $\times$ 128 grids $\times$ 30000 steps $\times$ 0.5, where 0.5 represents the volume fraction of γ'), and using PyCalphad, this would take 2.3 years (0.30 s $\times$ 2.5×10^8 times).

Supplementary Note 2: Detailed derivation of the local minimization condition

The local Gibbs free energy of the microstructure f_{local} is calculated as follows:

$$f_{local} = \sum_{\alpha=1}^{N} \phi_\alpha f_\alpha. \tag{S1}$$

To satisfy the constraints of Equations (1) and (2), we define the Lagrangian function L as follows:

$$L = f_{local} + \sum_{i=1}^{n-1} \lambda^i \left(c^i - \sum_{\alpha=1}^{N} \phi_\alpha \sum_{a \in \alpha} S_a y_a^i \right), \tag{S2}$$

where λ^i is a Lagrange multiplier. The condition that minimizes the f_{local} while satisfying Equations (1) and (2) is derived as follows:

$$\frac{\partial L}{\partial y_a^i} = \phi_\alpha \frac{\partial f_\alpha}{\partial y_a^i} - \lambda^i \phi_\alpha S_a = 0 \tag{S3}$$

$$\therefore \lambda^i = \frac{1}{S_a} \frac{\partial f_\alpha}{\partial y_a^i}. \tag{S4}$$

Therefore, the following relationship is obtained between sublattice a in phase α and sublattice b in phase β.

$$\frac{1}{S_a} \frac{\partial f_\alpha}{\partial y_a^i} = \frac{1}{S_b} \frac{\partial f_\beta}{\partial y_b^i} \tag{S5}$$

Supplementary Note 3: Detailed derivation of governing equations

Using the finite difference method, the changes in the site fraction of component i in sublattices a and b are denoted as Δy_a^i and Δy_b^i, respectively. The ratio of Δy_a^i to Δy_b^i is expressed as k_{ba}^i, as follows:

$$\Delta y_b^i = k_{ba}^i \Delta y_a^i. \tag{S6}$$

Therefore, the site fractions of component i in sublattices a and b after timestep Δt can be expressed as follows:

$$y_a^i\big|_{t+\Delta t} = y_a^i\big|_t + \Delta y_a^i \tag{S7}$$

$$y_b^i\big|_{t+\Delta t} = y_b^i\big|_t + k_{ba}^i \Delta y_a^i. \tag{S8}$$

Equations (S7) and (S8) yield the following equations:

$$\frac{\partial y_a^i}{\partial \Delta y_a^i} = 1 \tag{S9}$$

$$\frac{\partial y_b^i}{\partial \Delta y_a^i} = k_{ba}^i. \tag{S10}$$

We define the diffusion potential difference function H^i, which corresponds to the difference between the right and left sides of Equation (3) after changes in the site fraction, as follows:

$$H^i(\Delta y_a^1, \dots, \Delta y_a^{n-1}) = \frac{1}{S_a}\frac{\partial f_\alpha}{\partial y_a^i}(y_a^1 + \Delta y_a^1, \dots, y_a^{n-1} + \Delta y_a^{n-1}) - \frac{1}{S_b}\frac{\partial f_\beta}{\partial y_b^i}(y_b^1 + k_{ba}^1 \Delta y_a^1, \dots, y_b^{n-1} + k_{ba}^{n-1}\Delta y_a^{n-1}). \tag{S11}$$

The Maclaurin expansion of Equation (S11) is

$$H^i(\Delta y_a^1, \dots, \Delta y_a^{n-1}) = \sum_{k=0}^{\infty} \frac{1}{k!}\left(\Delta y_a^1 \frac{\partial}{\partial \Delta y_a^1} + \cdots + \Delta y_a^{n-1}\frac{\partial}{\partial \Delta y_a^{n-1}}\right)^k H^i(0, \dots, 0)$$

$$= \sum_{k=0}^{\infty} \frac{1}{k!}\left(\Delta y_a^1 \frac{\partial}{\partial \Delta y_a^1} + \cdots + \Delta y_a^{n-1}\frac{\partial}{\partial \Delta y_a^{n-1}}\right)^k \left(\frac{1}{S_a}\frac{\partial f_\alpha}{\partial y_a^i} - \frac{1}{S_b}\frac{\partial f_\beta}{\partial y_b^i}\right). \tag{S12}$$

The change in the site fraction per time step (Δy) is sufficiently small such that terms of $(\Delta y)^2$ and higher can be ignored. Ignoring the second and higher orders of k, Equation (S12) can be rewritten as follows:

$$H^i(\Delta y_a^1, \dots, \Delta y_a^{n-1}) = \frac{1}{S_a}\frac{\partial f_\alpha}{\partial y_a^i} - \frac{1}{S_b}\frac{\partial f_\beta}{\partial y_b^i} + \left(\Delta y_a^1 \frac{\partial}{\partial \Delta y_a^1} + \cdots + \Delta y_a^{n-1}\frac{\partial}{\partial \Delta y_a^{n-1}}\right)\left(\frac{1}{S_a}\frac{\partial f_\alpha}{\partial y_a^i} - \frac{1}{S_b}\frac{\partial f_\beta}{\partial y_b^i}\right). \tag{S13}$$

When the cross terms are overestimated by substituting the diagonal term into the cross terms, Equation (S13) is expressed as follows:

$$H^i(\Delta y_a^1, \dots, \Delta y_a^{n-1}) = \frac{1}{S_a}\frac{\partial f_\alpha}{\partial y_a^i} - \frac{1}{S_b}\frac{\partial f_\beta}{\partial y_b^i} + (n-1)\Delta y_a^i \frac{\partial}{\partial \Delta y_a^i}\left(\frac{1}{S_a}\frac{\partial f_\alpha}{\partial y_a^i} - \frac{1}{S_b}\frac{\partial f_\beta}{\partial y_b^i}\right). \tag{S14}$$

This overestimation ensures that the site fractions change in order to achieve the local minimization condition and that the calculations do not diverge. By applying the chain rule and Equations (S9) and (S10) to Equation (S14), we obtain

$$H^i(\Delta y_a^1, \dots, \Delta y_a^{n-1}) = \frac{1}{S_a}\frac{\partial f_\alpha}{\partial y_a^i} - \frac{1}{S_b}\frac{\partial f_\beta}{\partial y_b^i} + (n-1)\Delta y_a^i \frac{\partial y_a^i}{\partial \Delta y_a^i}\frac{\partial}{\partial y_a^i}\frac{1}{S_a}\frac{\partial f_\alpha}{\partial y_a^i} - (n-1)\Delta y_b^i \frac{\partial y_b^i}{\partial \Delta y_b^i}\frac{\partial}{\partial y_b^i}\frac{1}{S_b}\frac{\partial f_\beta}{\partial y_b^i}$$
$$= \frac{1}{S_a}\frac{\partial f_\alpha}{\partial y_a^i} - \frac{1}{S_b}\frac{\partial f_\beta}{\partial y_b^i} + (n-1)\Delta y_a^i \frac{\partial}{\partial y_a^i}\frac{1}{S_a}\frac{\partial f_\alpha}{\partial y_a^i} - (n-1)\Delta y_a^i k_{ba}^i \frac{\partial}{\partial y_b^i}\frac{1}{S_b}\frac{\partial f_\beta}{\partial y_b^i}. \tag{S15}$$

Considering the local minimization condition ($H^i = 0$), Equation (S15) yields

$$k_{ba}^i = \frac{\frac{1}{S_a}\frac{\partial f_\alpha}{\partial y_a^i} - \frac{1}{S_b}\frac{\partial f_\beta}{\partial y_b^i} + (n-1)\Delta y_a^i \frac{1}{S_a}\frac{\partial^2 f_a}{\left(\partial y_a^i\right)^2}}{(n-1)\Delta y_a^i \frac{1}{S_b}\frac{\partial^2 f_\beta}{\left(\partial y_b^i\right)^2}}. \tag{S16}$$

The two necessary conditions for the phase-field model—solute diffusion in the bulk phase and conservation of the solute around the moving interface—can be expressed as follows:

$$\sum_{\beta=1}^{N} \phi_\beta \sum_{b\in\beta} S_b y_b^i + \frac{\partial c^i}{\partial t}\Delta t = \sum_{\beta=1}^{N}\left(\phi_\beta + \frac{\partial \phi_\beta}{\partial t}\Delta t\right)\sum_{b\in\beta} S_b\left(y_b^i + k_{ba}^i \Delta y_a^i\right). \tag{S17}$$

Substituting Equation (S16) into Equation (S17) yields the following equation:

$$\Delta y_a^i = \frac{-\sum_{\beta=1}^{N}\left(\phi_\beta + \frac{\partial \phi_\beta}{\partial t}\Delta t\right)\sum_{b\in\beta} S_b\left(\frac{1}{S_a}\frac{\partial f_\alpha}{\partial y_a^i} - \frac{1}{S_b}\frac{\partial f_\beta}{\partial y_b^i}\right)\Big/\left(\frac{n-1}{S_b}\frac{\partial^2 f_\beta}{\left(\partial y_b^i\right)^2}\right) + \left(\frac{\partial c^i}{\partial t} - \sum_{b\in\beta}\frac{\partial \phi_\beta}{\partial t}\sum_b^M S_b y_b^i\right)\Delta t}{\sum_{\beta=1}^{N}\left(\phi_\beta + \frac{\partial \phi_\beta}{\partial t}\Delta t\right)\sum_{b\in\beta}\frac{S_b^2}{S_a}\frac{\partial^2 f_\alpha}{\left(\partial y_a^i\right)^2}\Big/\frac{\partial^2 f_\beta}{\left(\partial y_b^i\right)^2}}. \tag{S18}$$

Supplementary Note 4: Detailed discussion of the stability conditions

Eqs. (S14) – (S18) were derived by substituting the diagonal term into the cross terms in Eq. (S13). This substitution corresponds to the overestimation of the cross terms because, in general, cross terms in the thermodynamic factor has less influence on calculations compared to diagonal terms and is therefore often neglected. Here, as a contrasting case, the situation where the cross terms are underestimated is considered. By neglecting the cross terms in Eq. (S13), the following equation can be obtained:

$$H^i(\Delta y_a^1, \dots, \Delta y_a^{n-1}) = \frac{1}{S_a}\frac{\partial f_\alpha}{\partial y_a^i} - \frac{1}{S_b}\frac{\partial f_\beta}{\partial y_b^i} + \Delta y_a^i \frac{\partial}{\partial \Delta y_a^i}\left(\frac{1}{S_a}\frac{\partial f_\alpha}{\partial y_a^i} - \frac{1}{S_b}\frac{\partial f_\beta}{\partial y_b^i}\right). \tag{S19}$$

By applying the chain rule and Eqs. (S9) and (S10) to Eq. (S19), the following equation can be obtained

$$H^i(\Delta y_a^1, \dots, \Delta y_a^{n-1}) = \frac{1}{S_a}\frac{\partial f_\alpha}{\partial y_a^i} - \frac{1}{S_b}\frac{\partial f_\beta}{\partial y_b^i} + \Delta y_a^i \frac{\partial y_a^i}{\partial \Delta y_a^i}\frac{\partial}{\partial y_a^i}\frac{1}{S_a}\frac{\partial f_\alpha}{\partial y_a^i} - \Delta y_b^i \frac{\partial y_b^i}{\partial \Delta y_b^i}\frac{\partial}{\partial y_b^i}\frac{1}{S_b}\frac{\partial f_\beta}{\partial y_b^i}$$
$$= \frac{1}{S_a}\frac{\partial f_\alpha}{\partial y_a^i} - \frac{1}{S_b}\frac{\partial f_\beta}{\partial y_b^i} + \Delta y_a^i \frac{\partial}{\partial y_a^i}\frac{1}{S_a}\frac{\partial f_\alpha}{\partial y_a^i} - \Delta y_a^i k_{ba}^i \frac{\partial}{\partial y_b^i}\frac{1}{S_b}\frac{\partial f_\beta}{\partial y_b^i}. \tag{S20}$$

Considering the local minimization condition ($H^i = 0$), Eq. (S20) yields

$$k_{ba}^{i}=\frac{\frac{1}{S_a}\frac{\partial f_\alpha}{\partial y_a^i}-\frac{1}{S_b}\frac{\partial f_\beta}{\partial y_b^i}+\Delta y_a^i\frac{1}{S_a}\frac{\partial^2 f_a}{\left(\partial y_a^i\right)^2}}{\Delta y_a^i\frac{1}{S_b}\frac{\partial^2 f_\beta}{\left(\partial y_b^i\right)^2}}. \tag{S21}$$

Substituting Eq. (S21) into Eq. (S17) yields the following equation:

$$\Delta y_a^i=\frac{-\sum_{\beta=1}^{N}\left(\phi_\beta+\frac{\partial\phi_\beta}{\partial t}\Delta t\right)\sum_{b\in\beta}S_b\left(\frac{1}{S_a}\frac{\partial f_\alpha}{\partial y_a^i}-\frac{1}{S_b}\frac{\partial f_\beta}{\partial y_b^i}\right)\Big/\left(\frac{1}{S_b}\frac{\partial^2 f_\beta}{\left(\partial y_b^i\right)^2}\right)+\left(\frac{\partial c^i}{\partial t}-\sum_{b\in\beta}\frac{\partial\phi_\beta}{\partial t}\sum_b^M S_b y_b^i\right)\Delta t}{\sum_{\beta=1}^{N}\left(\phi_\beta+\frac{\partial\phi_\beta}{\partial t}\Delta t\right)\sum_{b\in\beta}\frac{S_b^2}{S_a}\frac{\partial^2 f_\alpha}{(\partial y_a^i)^2}\Big/\frac{\partial^2 f_\beta}{\left(\partial y_b^i\right)^2}}. \tag{S22}$$

Eqs. (S18) and (S22) are obtained by overestimating and underestimating the cross terms in Eq. (S13), respectively. Therefore, when the cross terms are evaluated without estimation, the evolution equation of the site fraction can be regarded as lying between the two contrasting cases as follows:

$$\Delta y_a^i=\frac{-\sum_{\beta=1}^{N}\left(\phi_\beta+\frac{\partial\phi_\beta}{\partial t}\Delta t\right)\sum_{b\in\beta}S_b\left(\frac{1}{S_a}\frac{\partial f_\alpha}{\partial y_a^i}-\frac{1}{S_b}\frac{\partial f_\beta}{\partial y_b^i}\right)\Big/\left(\frac{A}{S_b}\frac{\partial^2 f_\beta}{\left(\partial y_b^i\right)^2}\right)+\left(\frac{\partial c^i}{\partial t}-\sum_{b\in\beta}\frac{\partial\phi_\beta}{\partial t}\sum_b^M S_b y_b^i\right)\Delta t}{\sum_{\beta=1}^{N}\left(\phi_\beta+\frac{\partial\phi_\beta}{\partial t}\Delta t\right)\sum_{b\in\beta}\frac{S_b^2}{S_a}\frac{\partial^2 f_\alpha}{\left(\partial y_a^i\right)^2}\Big/\frac{\partial^2 f_\beta}{\left(\partial y_b^i\right)^2}}, \tag{S23}$$

where A is a constant between 1 (in the case of underestimation) and $n-1$ (in the case of the overestimation). Since the divergence of the calculation arises due to the excessive change in the site fraction, the calculation should be performed under the condition where the first term of the numerator of Eq. (S23) is minimized as follows:

$$\Delta y_a^i=\frac{-\sum_{\beta=1}^{N}\left(\phi_\beta+\frac{\partial\phi_\beta}{\partial t}\Delta t\right)\sum_{b\in\beta}S_b\left(\frac{1}{S_a}\frac{\partial f_\alpha}{\partial y_a^i}-\frac{1}{S_b}\frac{\partial f_\beta}{\partial y_b^i}\right)\Big/\left(\frac{\max(A)}{S_b}\frac{\partial^2 f_\beta}{\left(\partial y_b^i\right)^2}\right)+\left(\frac{\partial c^i}{\partial t}-\sum_{b\in\beta}\frac{\partial\phi_\beta}{\partial t}\sum_b^M S_b y_b^i\right)\Delta t}{\sum_{\beta=1}^{N}\left(\phi_\beta+\frac{\partial\phi_\beta}{\partial t}\Delta t\right)\sum_{b\in\beta}\frac{S_b^2}{S_a}\frac{\partial^2 f_\alpha}{\left(\partial y_a^i\right)^2}\Big/\frac{\partial^2 f_\beta}{\left(\partial y_b^i\right)^2}}, \tag{S24}$$

$$\therefore \Delta y_a^i=\frac{-\sum_{\beta=1}^{N}\left(\phi_\beta+\frac{\partial\phi_\beta}{\partial t}\Delta t\right)\sum_{b\in\beta}S_b\left(\frac{1}{S_a}\frac{\partial f_\alpha}{\partial y_a^i}-\frac{1}{S_b}\frac{\partial f_\beta}{\partial y_b^i}\right)\Big/\left(\frac{n-1}{S_b}\frac{\partial^2 f_\beta}{\left(\partial y_b^i\right)^2}\right)+\left(\frac{\partial c^i}{\partial t}-\sum_{b\in\beta}\frac{\partial\phi_\beta}{\partial t}\sum_b^M S_b y_b^i\right)\Delta t}{\sum_{\beta=1}^{N}\left(\phi_\beta+\frac{\partial\phi_\beta}{\partial t}\Delta t\right)\sum_{b\in\beta}\frac{S_b^2}{S_a}\frac{\partial^2 f_\alpha}{\left(\partial y_a^i\right)^2}\Big/\frac{\partial^2 f_\beta}{\left(\partial y_b^i\right)^2}}. \tag{S25}$$

Eq. (S25) correspond to the equation when the cross terms of Eq. (S13) are overestimated. Therefore, the overestimation ensures that the calculations do not diverge.

Supplementary Note 5: Proof that the governing equation of Δy_a^i can be reduced to the standard governing equation of composition

The change in composition per step Δc can be expressed using Equation (7) as follows:

$$\Delta c=c^i\big|_{t+\Delta t}-c^i\big|_t$$

$$= \sum_{\alpha}^{N} \left(\phi_\alpha + \frac{\partial \phi_\alpha}{\partial t} \cdot \Delta t\right) \sum_{a \in \alpha} S_a \left(y_a^i + \Delta y_a^i\right) - \sum_{\alpha}^{N} \phi_\alpha \sum_{a \in \alpha} S_a y_a^i$$

$$= \sum_{\alpha}^{N} \left(\phi_\alpha + \frac{\partial \phi_\alpha}{\partial t} \Delta t\right) \sum_{a \in \alpha} S_a \left[y_a^i + \frac{-\sum_{\beta=1}^{N} \left(\phi_\beta + \frac{\partial \phi_\beta}{\partial t} \Delta t\right) \sum_{b \in \beta} S_b \left(\frac{1}{S_a} \frac{\partial f_\alpha}{\partial y_a^i} - \frac{1}{S_b} \frac{\partial f_\beta}{\partial y_b^i}\right) \Big/ \left(\frac{n-1}{S_b} \frac{\partial^2 f_\beta}{\left(\partial y_b^i\right)^2}\right) + \left(\frac{\partial c^i}{\partial t} - \sum_{b \in \beta} \frac{\partial \phi_\beta}{\partial t} \sum_b^M S_b y_b^i\right) \Delta t}{\sum_{\beta=1}^{N} \left(\phi_\beta + \frac{\partial \phi_\beta}{\partial t} \Delta t\right) \sum_{b \in \beta} \frac{S_b^2}{S_a} \frac{\partial^2 f_\alpha}{\left(\partial y_a^i\right)^2} \Big/ \frac{\partial^2 f_\beta}{\left(\partial y_b^i\right)^2}} \right] - \sum_{\alpha}^{N} \phi_\alpha \sum_{a \in \alpha} S_a y_a^i$$

$$= \sum_{\alpha}^{N} \left(\phi_\alpha + \frac{\partial \phi_\alpha}{\partial t} \Delta t\right) \sum_{a \in \alpha} S_a \left[\frac{-\sum_{\beta=1}^{N} \left(\phi_\beta + \frac{\partial \phi_\beta}{\partial t} \Delta t\right) \sum_{b \in \beta} S_b \left(\frac{1}{S_a} \frac{\partial f_\alpha}{\partial y_a^i} - \frac{1}{S_b} \frac{\partial f_\beta}{\partial y_b^i}\right) \Big/ \left(\frac{n-1}{S_b} \frac{\partial^2 f_\beta}{\left(\partial y_b^i\right)^2}\right)}{\sum_{\beta=1}^{N} \left(\phi_\beta + \frac{\partial \phi_\beta}{\partial t} \Delta t\right) \sum_{b \in \beta} \frac{S_b^2}{S_a} \frac{\partial^2 f_\alpha}{\left(\partial y_a^i\right)^2} \Big/ \frac{\partial^2 f_\beta}{\left(\partial y_b^i\right)^2}} \right] + \sum_{\alpha}^{N} \left(\phi_\alpha + \frac{\partial \phi_\alpha}{\partial t} \Delta t\right) \sum_{a \in \alpha} S_a \left[y_a^i + \frac{\left(\frac{\partial c^i}{\partial t} - \sum_{b \in \beta} \frac{\partial \phi_\beta}{\partial t} \sum_b^M S_b y_b^i\right) \Delta t}{\sum_{\beta=1}^{N} \left(\phi_\beta + \frac{\partial \phi_\beta}{\partial t} \Delta t\right) \sum_{b \in \beta} \frac{S_b^2}{S_a} \frac{\partial^2 f_\alpha}{\left(\partial y_a^i\right)^2} \Big/ \frac{\partial^2 f_\beta}{\left(\partial y_b^i\right)^2}} \right] - \sum_{\alpha}^{N} \phi_\alpha \sum_{a \in \alpha} S_a y_a^i$$

$$= A + B. \tag{S26}$$

Here, we define A and B as follows:

$$A = \sum_{\alpha}^{N} \left(\phi_\alpha + \frac{\partial \phi_\alpha}{\partial t} \Delta t\right) \sum_{a \in \alpha} S_a \left[\frac{-\sum_{\beta=1}^{N} \left(\phi_\beta + \frac{\partial \phi_\beta}{\partial t} \Delta t\right) \sum_{b \in \beta} S_b \left(\frac{1}{S_a} \frac{\partial f_\alpha}{\partial y_a^i} - \frac{1}{S_b} \frac{\partial f_\beta}{\partial y_b^i}\right) \Big/ \left(\frac{n-1}{S_b} \frac{\partial^2 f_\beta}{\left(\partial y_b^i\right)^2}\right)}{\sum_{\beta=1}^{N} \left(\phi_\beta + \frac{\partial \phi_\beta}{\partial t} \Delta t\right) \sum_{b \in \beta} \frac{S_b^2}{S_a} \frac{\partial^2 f_\alpha}{\left(\partial y_a^i\right)^2} \Big/ \frac{\partial^2 f_\beta}{\left(\partial y_b^i\right)^2}} \right] \tag{S27}$$

$$B = \sum_{\alpha}^{N} \left(\phi_\alpha + \frac{\partial \phi_\alpha}{\partial t} \Delta t\right) \sum_{a \in \alpha} S_a \left[y_a^i + \frac{\left(\frac{\partial c^i}{\partial t} - \sum_{b \in \beta} \frac{\partial \phi_\beta}{\partial t} \sum_b^M S_b y_b^i\right) \Delta t}{\sum_{\beta=1}^{N} \left(\phi_\beta + \frac{\partial \phi_\beta}{\partial t} \Delta t\right) \sum_{b \in \beta} \frac{S_b^2}{S_a} \frac{\partial^2 f_\alpha}{\left(\partial y_a^i\right)^2} \Big/ \frac{\partial^2 f_\beta}{\left(\partial y_b^i\right)^2}} \right] - \sum_{\alpha}^{N} \phi_\alpha \sum_{a \in \alpha} S_a y_a^i. \tag{S28}$$

A can be calculated as follows:

$$A = \sum_{\alpha}^{N} \left(\phi_\alpha + \frac{\partial \phi_\alpha}{\partial t} \Delta t\right) \sum_{a \in \alpha} S_a \left[\frac{-\sum_{\beta=1}^{N} \left(\phi_\beta + \frac{\partial \phi_\beta}{\partial t} \Delta t\right) \sum_{b \in \beta} S_b \left(\frac{1}{S_a} \frac{\partial f_\alpha}{\partial y_a^i} - \frac{1}{S_b} \frac{\partial f_\beta}{\partial y_b^i}\right) \Big/ \left(\frac{n-1}{S_b} \frac{\partial^2 f_\beta}{\left(\partial y_b^i\right)^2}\right)}{\sum_{\beta=1}^{N} \left(\phi_\beta + \frac{\partial \phi_\beta}{\partial t} \Delta t\right) \sum_{b \in \beta} \frac{S_b^2}{S_a} \frac{\partial^2 f_\alpha}{(\partial y_a^i)^2} \Big/ \frac{\partial^2 f_\beta}{\left(\partial y_b^i\right)^2}} \right]$$

$$= \sum_{\alpha}^{N} \left(\phi_\alpha + \frac{\partial \phi_\alpha}{\partial t} \Delta t\right) \sum_{a \in \alpha} S_a \left[\frac{-\sum_{\beta=1}^{N} \left(\phi_\beta + \frac{\partial \phi_\beta}{\partial t} \Delta t\right) \sum_{b \in \beta} S_b \left(\frac{1}{S_a} \frac{\partial f_\alpha}{\partial y_a^i}\right) \Big/ \left(\frac{n-1}{S_b} \frac{\partial^2 f_\beta}{\left(\partial y_b^i\right)^2}\right)}{\sum_{\beta=1}^{N} \left(\phi_\beta + \frac{\partial \phi_\beta}{\partial t} \Delta t\right) \sum_{b \in \beta} \frac{S_b^2}{S_a} \frac{\partial^2 f_\alpha}{\left(\partial y_a^i\right)^2} \Big/ \frac{\partial^2 f_\beta}{\left(\partial y_b^i\right)^2}} \right] + \sum_{\alpha}^{N} \left(\phi_\alpha + \frac{\partial \phi_\alpha}{\partial t} \Delta t\right) \sum_{a \in \alpha} S_a \left[\frac{-\sum_{\beta=1}^{N} \left(\phi_\beta + \frac{\partial \phi_\beta}{\partial t} \Delta t\right) \sum_{b \in \beta} S_b \left(-\frac{1}{S_b} \frac{\partial f_\beta}{\partial y_b^i}\right) \Big/ \left(\frac{n-1}{S_b} \frac{\partial^2 f_\beta}{\left(\partial y_b^i\right)^2}\right)}{\sum_{\beta=1}^{N} \left(\phi_\beta + \frac{\partial \phi_\beta}{\partial t} \Delta t\right) \sum_{b \in \beta} \frac{S_b^2}{S_a} \frac{\partial^2 f_\alpha}{\left(\partial y_a^i\right)^2} \Big/ \frac{\partial^2 f_\beta}{\left(\partial y_b^i\right)^2}} \right]$$

$$= A' + A''. \tag{S29}$$

Here, we define A' and A'' as follows:

$$A' = \sum_{\alpha}^{N} \left(\phi_\alpha + \frac{\partial \phi_\alpha}{\partial t} \Delta t\right) \sum_{a \in \alpha} S_a \left[\frac{-\sum_{\beta=1}^{N} \left(\phi_\beta + \frac{\partial \phi_\beta}{\partial t} \Delta t\right) \sum_{b \in \beta} S_b \left(\frac{1}{S_a} \frac{\partial f_\alpha}{\partial y_a^i}\right) \Big/ \left(\frac{n-1}{S_b} \frac{\partial^2 f_\beta}{\left(\partial y_b^i\right)^2}\right)}{\sum_{\beta=1}^{N} \left(\phi_\beta + \frac{\partial \phi_\beta}{\partial t} \Delta t\right) \sum_{b \in \beta} \frac{S_b^2}{S_a} \frac{\partial^2 f_\alpha}{(\partial y_a^i)^2} \Big/ \frac{\partial^2 f_\beta}{\left(\partial y_b^i\right)^2}} \right] \tag{S30}$$

$$A'' = \sum_{\alpha}^{N} \left(\phi_\alpha + \frac{\partial \phi_\alpha}{\partial t} \Delta t\right) \sum_{a \in \alpha} S_a \left[\frac{-\sum_{\beta=1}^{N} \left(\phi_\beta + \frac{\partial \phi_\beta}{\partial t} \Delta t\right) \sum_{b \in \beta} S_b \left(-\frac{1}{S_b} \frac{\partial f_\beta}{\partial y_b^i}\right) \Big/ \left(\frac{n-1}{S_b} \frac{\partial^2 f_\beta}{\left(\partial y_b^i\right)^2}\right)}{\sum_{\beta=1}^{N} \left(\phi_\beta + \frac{\partial \phi_\beta}{\partial t} \Delta t\right) \sum_{b \in \beta} \frac{S_b^2}{S_a} \frac{\partial^2 f_\alpha}{(\partial y_a^i)^2} \Big/ \frac{\partial^2 f_\beta}{\left(\partial y_b^i\right)^2}} \right]. \tag{S31}$$

A' can be calculated as follows:

$$A' = \frac{\sum_{\beta=1}^{N} \left(\phi_\beta + \frac{\partial \phi_\beta}{\partial t} \Delta t\right) \sum_{b \in \beta} S_b \Big/ \left(\frac{1}{S_b} \frac{\partial^2 f_\beta}{\left(\partial y_b^i\right)^2}\right)}{\sum_{\beta=1}^{N} \left(\phi_\beta + \frac{\partial \phi_\beta}{\partial t} \Delta t\right) \sum_{b \in \beta} S_b^2 \Big/ \frac{\partial^2 f_\beta}{\left(\partial y_b^i\right)^2}} \sum_{\alpha}^{N} \left(\phi_\alpha + \frac{\partial \phi_\alpha}{\partial t} \Delta t\right) \sum_{a \in \alpha} S_a \left[\frac{-\frac{1}{S_a} \frac{\partial f_\alpha}{\partial y_a^i}}{\frac{n-1}{S_a} \frac{\partial^2 f_\alpha}{(\partial y_a^i)^2}} \right]$$

$$
= \sum_{\alpha}^{N} \left(\phi_\alpha + \frac{\partial \phi_\alpha}{\partial t} \Delta t \right) \sum_{a \in \alpha} S_a \left[\frac{-\frac{1}{S_a} \frac{\partial f_\alpha}{\partial y_a^i}}{\frac{n-1}{S_a} \frac{\partial^2 f_\alpha}{(\partial y_a^i)^2}} \right]. \quad \text{(S32)}
$$

A'' can be calculated as follows:

$$
A'' = \frac{-\sum_{\beta=1}^{N} \left(\phi_\beta + \frac{\partial \phi_\beta}{\partial t} \Delta t \right) \sum_{b \in \beta} S_b \left(-\frac{1}{S_b} \frac{\partial f_\beta}{\partial y_b^i} \right) \Big/ \left(\frac{n-1}{S_b} \frac{\partial^2 f_\beta}{\left(\partial y_b^i\right)^2} \right)}{\sum_{\beta=1}^{N} \left(\phi_\beta + \frac{\partial \phi_\beta}{\partial t} \Delta t \right) \sum_{b \in \beta} S_b^2 \Big/ \frac{\partial^2 f_\beta}{\left(\partial y_b^i\right)^2}} \sum_{\alpha}^{N} \left(\phi_\alpha + \frac{\partial \phi_\alpha}{\partial t} \Delta t \right) \sum_{a \in \alpha} S_a \left[\frac{1}{\frac{1}{S_a} \frac{\partial^2 f_\alpha}{\left(\partial y_a^i\right)^2}} \right]
$$

$$
= \frac{-\sum_{\beta=1}^{N} \left(\phi_\beta + \frac{\partial \phi_\beta}{\partial t} \Delta t \right) \sum_{b \in \beta} S_b \left(-\frac{1}{S_b} \frac{\partial f_\beta}{\partial y_b^i} \right) \Big/ \left(\frac{n-1}{S_b} \frac{\partial^2 f_\beta}{\left(\partial y_b^i\right)^2} \right)}{\sum_{\beta=1}^{N} \left(\phi_\beta + \frac{\partial \phi_\beta}{\partial t} \Delta t \right) \sum_{b \in \beta} S_b^2 \Big/ \frac{\partial^2 f_\beta}{\left(\partial y_b^i\right)^2}} \sum_{\alpha}^{N} \left(\phi_\alpha + \frac{\partial \phi_\alpha}{\partial t} \Delta t \right) \sum_{a \in \alpha} S_a^2 \Big/ \frac{\partial^2 f_\alpha}{\left(\partial y_a^i\right)^2}
$$

$$
= -\sum_{\beta=1}^{N} \left(\phi_\beta + \frac{\partial \phi_\beta}{\partial t} \Delta t \right) \sum_{b \in \beta} S_b \left(-\frac{1}{S_b} \frac{\partial f_\beta}{\partial y_b^i} \right) \Big/ \left(\frac{n-1}{S_b} \frac{\partial^2 f_\beta}{\left(\partial y_b^i\right)^2} \right). \quad \text{(S33)}
$$

Equations (S29), (S32), and (S33) yield

$$
A = A' + A'' = \sum_{\alpha}^{N} \left(\phi_\alpha + \frac{\partial \phi_\alpha}{\partial t} \Delta t \right) \sum_{a \in \alpha} S_a \left[\frac{-\frac{1}{S_a} \frac{\partial f_\alpha}{\partial y_a^i}}{\frac{n-1}{S_a} \frac{\partial^2 f_\alpha}{\left(\partial y_a^i\right)^2}} \right] - \sum_{\beta=1}^{N} \left(\phi_\beta + \frac{\partial \phi_\beta}{\partial t} \Delta t \right) \sum_{b \in \beta} S_b \left(-\frac{1}{S_b} \frac{\partial f_\beta}{\partial y_b^i} \right) \Big/ \left(\frac{n-1}{S_b} \frac{\partial^2 f_\beta}{\left(\partial y_b^i\right)^2} \right) = 0 \quad \text{(S34)}.
$$

B can be calculated as follows:

$$
B = \sum_{\alpha}^{N} \left(\phi_\alpha + \frac{\partial \phi_\alpha}{\partial t} \Delta t \right) \sum_{a \in \alpha} S_a \left[y_a^i + \frac{\left(\frac{\partial c^i}{\partial t} - \sum_{b \in \beta} \frac{\partial \phi_\beta}{\partial t} \sum_b^M S_b y_b^i \right) \Delta t}{\sum_{\beta=1}^{N} \left(\phi_\beta + \frac{\partial \phi_\beta}{\partial t} \Delta t \right) \sum_{b \in \beta} \frac{S_b^2}{S_a} \frac{\partial^2 f_\alpha}{\left(\partial y_a^i\right)^2} \Big/ \frac{\partial^2 f_\beta}{\left(\partial y_b^i\right)^2}} \right] - \sum_{\alpha}^{N} \phi_\alpha \sum_{a \in \alpha} S_a y_a^i
$$

$$
= \sum_{\alpha}^{N} \frac{\partial \phi_\alpha}{\partial t} \Delta t \sum_{a \in \alpha} S_a y_a^i + \sum_{\alpha}^{N} \left(\phi_\alpha + \frac{\partial \phi_\alpha}{\partial t} \Delta t \right) \sum_{a \in \alpha} S_a \frac{\left(\frac{\partial c^i}{\partial t} - \sum_{b \in \beta} \frac{\partial \phi_\beta}{\partial t} \sum_b^M S_b y_b^i \right) \Delta t}{\sum_{\beta=1}^{N} \left(\phi_\beta + \frac{\partial \phi_\beta}{\partial t} \Delta t \right) \sum_{b \in \beta} \frac{S_b^2}{S_a} \frac{\partial^2 f_\alpha}{\left(\partial y_a^i\right)^2} \Big/ \frac{\partial^2 f_\beta}{\left(\partial y_b^i\right)^2}}
$$

$$
= \sum_{\alpha}^{N} \frac{\partial \phi_\alpha}{\partial t} \Delta t \sum_{a \in \alpha} S_a y_a^i + \sum_{\alpha}^{N} \left(\phi_\alpha + \frac{\partial \phi_\alpha}{\partial t} \Delta t \right) \sum_{a \in \alpha} \frac{S_a^2}{\frac{\partial^2 f_\alpha}{\left(\partial y_a^i\right)^2}} \frac{\left(\frac{\partial c^i}{\partial t} - \sum_{b \in \beta} \frac{\partial \phi_\beta}{\partial t} \sum_b^M S_b y_b^i \right) \Delta t}{\sum_{\beta=1}^{N} \left(\phi_\beta + \frac{\partial \phi_\beta}{\partial t} \Delta t \right) \sum_{b \in \beta} S_b^2 \Big/ \frac{\partial^2 f_\beta}{\left(\partial y_b^i\right)^2}}
$$

$$
= \frac{1}{\sum_{\beta=1}^{N} \left(\phi_\beta + \frac{\partial \phi_\beta}{\partial t} \Delta t \right) \sum_{b \in \beta} S_b^2 \Big/ \frac{\partial^2 f_\beta}{\left(\partial y_b^i\right)^2}} \left[\sum_{\beta=1}^{N} \left(\phi_\beta + \frac{\partial \phi_\beta}{\partial t} \Delta t \right) \sum_{b \in \beta} S_b^2 \Big/ \frac{\partial^2 f_\beta}{\left(\partial y_b^i\right)^2} \sum_{\alpha}^{N} \frac{\partial \phi_\alpha}{\partial t} \Delta t \sum_{a \in \alpha} S_a y_a^i + \sum_{\alpha}^{N} \left(\phi_\alpha + \frac{\partial \phi_\alpha}{\partial t} \Delta t \right) \sum_{a \in \alpha} \frac{S_a^2}{\frac{\partial^2 f_\alpha}{(\partial y_a^i)^2}} \left(\frac{\partial c^i}{\partial t} - \sum_{b \in \beta} \frac{\partial \phi_\beta}{\partial t} \sum_{b \in \beta} S_b y_b^i \right) \Delta t \right]
$$

$$
= \frac{1}{\sum_{\beta=1}^{N} \left(\phi_\beta + \frac{\partial \phi_\beta}{\partial t} \Delta t \right) \sum_{b \in \beta} S_b^2 \Big/ \frac{\partial^2 f_\beta}{\left(\partial y_b^i\right)^2}} \left[\sum_{\beta=1}^{N} \left(\phi_\beta + \frac{\partial \phi_\beta}{\partial t} \Delta t \right) \sum_{b \in \beta} S_b^2 \Big/ \frac{\partial^2 f_\beta}{\left(\partial y_b^i\right)^2} \sum_{\alpha}^{N} \frac{\partial \phi_\alpha}{\partial t} \Delta t \sum_{a \in \alpha} S_a y_a^i + \sum_{\beta}^{N} \left(\phi_\beta + \frac{\partial \phi_\beta}{\partial t} \Delta t \right) \sum_{b \in \beta} \frac{S_a^2}{\frac{\partial^2 f_\alpha}{(\partial y_a^i)^2}} \left(\frac{\partial c^i}{\partial t} - \sum_{a \in \alpha} \frac{\partial \phi_\alpha}{\partial t} \sum_{a \in \alpha} S_a y_a^i \right) \Delta t \right]
$$

$$
= \frac{1}{\sum_{\beta=1}^{N} \left(\phi_\beta + \frac{\partial \phi_\beta}{\partial t} \Delta t \right) \sum_{b \in \beta} S_b^2 \Big/ \frac{\partial^2 f_\beta}{\left(\partial y_b^i\right)^2}} \left\{ \sum_{\beta=1}^{N} \left(\phi_\beta + \frac{\partial \phi_\beta}{\partial t} \Delta t \right) \sum_{b \in \beta} S_b^2 \Big/ \frac{\partial^2 f_\beta}{\left(\partial y_b^i\right)^2} \left[\sum_{\alpha}^{N} \frac{\partial \phi_\alpha}{\partial t} \Delta t \sum_{a \in \alpha} S_a y_a^i + \left(\frac{\partial c^i}{\partial t} - \sum_{a \in \alpha} \frac{\partial \phi_\alpha}{\partial t} \sum_{a \in \alpha} S_a y_a^i \right) \Delta t \right] \right\}
$$

$$= \sum_{\alpha}^{N} \frac{\partial \phi_\alpha}{\partial t} \Delta t \sum_{a \in \alpha} S_a y_a^i + \left(\frac{\partial c^i}{\partial t} - \sum_{a \in \alpha} \frac{\partial \phi_\alpha}{\partial t} \sum_{a \in \alpha} S_a y_a^i \right) \Delta t$$

$$= \frac{\partial c^i}{\partial t} \Delta t. \tag{S35}$$

Equations (S26), (S34), and (S35) yield

$$\Delta c = A + B = 0 + \frac{\partial c^i}{\partial t} \cdot \Delta t = \frac{\partial c^i}{\partial t} \cdot \Delta t. \tag{S36}$$

Therefore, Equation (7) can be reduced analytically to obtain a standard evolution equation for the composition.
